# A Paradigm Shift in Health Surveillance: Preparing for the Future of Longevity

**DOI:** 10.3389/ijph.2025.1607760

**Published:** 2025-04-28

**Authors:** Vivek Jason Jayaraj, Diane Woei-Quan Chong, Mohd Zulfadli Hafiz Bin Ismail, Nur Aisyah Abdul Rahim, Karthikeyanathan Ramoo

**Affiliations:** ^1^ Digital Health Division, Ministry of Health, Putrajaya, Malaysia; ^2^ Institute for Health Systems Research, National Institutes of Health, Ministry of Health, Setia Alam, Malaysia; ^3^ Sector for Biostatistics and Data Repository, National Institutes of Health, Ministry of Health, Setia Alam, Malaysia; ^4^ Department of Social and Preventive Medicine, Faculty of Medicine, Universiti Malaya, Kuala Lumpur, Malaysia; ^5^ Pejabat Kesihatan Daerah Gombak, Ministry of Health, Kuala Lumpur, Malaysia

**Keywords:** artificial intelligence, human machine interface, surveillance systems, digital health, internet of things

**The IJPH series “Young Researcher Editorial” is a training project of the Swiss School of Public Health.**

As lifespans lengthen, societies face the challenge of ensuring people live these additional years in good health [1]. The World Health Organization’s (WHO) Global Roadmap for Healthy Longevity envisions “years of good health approaching the biological life span, with physical, cognitive, and social functioning enabling wellbeing” [1, 2]. But as people age, their health problems become more complex, so we need to transform public health surveillance.

Traditional surveillance has depended on periodic surveys, hospital discharge data, and mortality statistics, which provide retrospective insights rather than data we can act on in real-time. In a digital era marked by personal computing, the internet, and mobile healthcare, surveillance must become proactive, continuous, and data-driven. We must proactively monitor and address the health concerns of our growing population of elderly citizens, harness technology to effectively address the complexities of ageing populations, and use big data analytics and artificial intelligence (AI) to enable predictive modelling, real-time insights, and personalised interventions. To future-proof our health systems we recommend leveraging technologies, big data analytics, and AI to create an adaptive surveillance ecosystem—one that evolves to integrate emerging technologies and novel data streams [1, 3].

In this essay, we explore two interlocking paradigm shifts: the first is a shift in foundational infrastructure, to centralised lifetime health records, ubiquitous IoT devices, and AI-driven analytics; the second is a shift in how we use advanced human–machine interfaces and responsive AI solutions to harness these real-time data streams for immediate interventions. The first shift will transform surveillance systems within a decade to address the health challenges of an ageing population. This shift depends on three components: centralised health records, ubiquitous IoT sensing for real-time monitoring, and AI-driven analytics for targeted interventions. Centralised lifetime health records (LHR) will become the primary repository for health data from birth to death, integrating electronic health records, genomic profiles, and social determinants of health [1, 4]. IoT sensing will collect and transmit health data in real-time to interconnected devices, such as smartwatches. Today, smartwatches can monitor heart rate, activity levels, sleep patterns, and even electrocardiograms; future iterations are likely to capture richer, more nuanced data about an individual’s health. A network of these sensors will overlay the LHR, continuously capturing real-time health data at individual and population levels [1]. IoT-infused LHR, alongside other non-traditional data from our environments and social media, will stream information to AIs and machine learning tools that will analyse data in real-time, identify patterns, predict outcomes, and detect emerging issues that might escape human observation [1]. This triad will facilitate precise interventions at population, community, and individual levels so we can prevent chronic conditions and personalise health strategies [1, 4, 5] extending health and life.

In alignment with the consequentialist paradigm of surveillance, the second shift will translate personalised surveillance data into immediate action via technologies like human-machine interfaces (HMIs) and AI [6]. An early example of an HMI is Neuralink, a chip that interfaces with the brain to assist individuals with neurological disorders. AIs of the future may be superintelligent agents that will constantly be available to us. In this scenario, advanced AIs will translate surveillance inputs from real-time IoT data and data developed in Phase 1 into real-time interventions implemented via HMIs, bypassing traditional intermediaries and improving the responsiveness of the public health system [7–9]. Imagine an older adult’s typical morning routine in this scenario. Upon waking, their AI companion reminds them to take their medication, having analysed their sleep patterns, vitals, and schedule for optimal timing and dosage. As they prepare for a bus ride, the AI detects subtle gait changes via sensors in their clothing and living space, advising them to slow down via a brain interface, and calculates an increased fall risk based on their current state. The AI guides them to a rest station to recuperate. Chemical and vitals sensors track their heart rate and blood sugar levels. As their vitals normalise and their gait improves, the system updates their status in real-time. Meanwhile, their AI/HMI assistant notifies emergency services to be on a yellow alert based on their location and health status. Anti-fall devices, like smart clothing, are primed for deployment if needed. While the first paradigm shift lays the groundwork, the second opens a new frontier in health surveillance and intervention, requiring health systems to be more modular and agile in adapting to evolving technologies and unforeseen challenges [1, 9, 10].

As we venture into this new era of health surveillance, we must navigate complex ethical challenges. Safeguarding against a “Big Brother” scenario is paramount, so data management systems must be robust and decentralised to prevent misuse of sensitive information [1, 5]. Participation in health surveillance should remain voluntary, based on informed consent. We must also ensure access to these technologies is equitable. We risk exacerbating health disparities if we fail to democratise access to devices and digital infrastructure [1, 2]. The digital divide extends beyond affordability, encompassing issues of connectivity, user-friendly design, and digital literacy. We must answer questions about data ownership, consent, and the right to be forgotten in lifelong health tracking. Our path forward demands technological innovation, moral imagination, and a sound ethical grounding, so health surveillance advancements serve the greater good without compromising fundamental rights [1]. We must also acknowledge and address the limitations of AI, such as biases embedded in algorithms, opaque decision-making processes, and the challenge of ensuring model accuracy across diverse populations. Government agencies must lead healthcare providers, technology companies, and academic institutions in collaborating to develop standards, protocols, and ethical frameworks for data collection and analysis. Public health professionals will need skills in data science, AI, and digital health to use these advanced surveillance systems [1, 5, 7, 10].

The future of health surveillance lies in the convergence of big data, IoT, AI, and advanced human-machine interfaces. By embracing these technologies and shifting our approach from reactive to proactive monitoring, we can better assure healthy longevity in an ageing world. As we navigate this transformation, we must remain committed to ethical principles and ensure the benefits of advanced health surveillance are distributed equitably across all segments of society. Only then can we truly realise the vision of a future where people of all ages can thrive, enjoy “ageing in place,” and reap the benefits of longer, healthier lives.

## Data Availability

The original contributions presented in the study are included in the article/supplementary material, further inquiries can be directed to the corresponding author.
